# Predictors of labor abnormalities in university hospital: unmatched case control study

**DOI:** 10.1186/1471-2393-14-256

**Published:** 2014-08-03

**Authors:** Wayu Abraham, Yifru Berhan

**Affiliations:** Hawassa University College of Medicine and Health Sciences, P.O.Box: 1560, Hawassa, Ethiopia

**Keywords:** “Case control”, “Labor abnormality”, “Ethiopia”

## Abstract

**Background:**

Abnormal labor is one of the common emergency obstetric problems contributing for more than two-thirds of the unplanned cesarean section. In Ethiopia, although labor abnormality and its complications like obstetric fistula are highly prevalent, there is no published study that determines the predictors of labor abnormalities.

**Methods:**

The study design was an unmatched case control which included 844 women (408 cases and 436 controls). Cases were identified when a woman was diagnosed to have one of the labor abnormalities at term (prolonged latent stage, active phase disorder, prolonged second stage, descent disorder and obstructed labor). Subgroup logistic regression analyses were done taking the different type of labor abnormalities as the dependent variable.

**Results:**

Nearly half of the cases (48.6%) were found to have the active phase disorder. Obstructed labor alone accounted for about 16.8% of the cases. The mean gestational age of cases and controls was almost comparable. More than a quarter of cases and controls came to the hospital in the second stage of labor. More than two-thirds of the cases (67.4%) gave birth by cesarean section. The logistic regression analysis demonstrated an independent association of overall labor abnormality with pelvic inadequacy. The subgroup analysis, however, revealed that several obstetric factors were associated with one or more types of labor abnormalities.

**Conclusion:**

Active phase disorders were the commonest type of labor abnormalities. Cases were late in reporting to the hospital. Malposition, inadequate pelvis and inadequate uterine contraction were some of the predictors of specific types of labor abnormalities.

## Background

Abnormal labor is one of the most common obstetric problems complicating about 20% of deliveries [1]. About 68% of unplanned cesarean section was reported to be due to the abnormal progress of labor among vertex presentations [2]. Labor abnormality can be encountered at all stages of labor as a prolonged latent first stage, active first stage disorder (protracted or arrested cervical dilatation or descent) and second stage disorder (prolonged or arrest of descent) [1]. To the extreme, labor is diagnosed as obstructed when the presenting part of the fetus could not descend and remains stuck for a long period of time in the birth canal despite adequate uterine contractions [3, 4].

The incidence of prolonged latent phase in spontaneously laboring women is reported to be 4 to 7 percent [5–7]. Women with prolonged latent phase of labor are at a higher risk of developing other types of labor abnormalities, and of requiring cesarean delivery more often, while their newborns are more likely to require neonatal intensive care unit admission [1, 7, 8]. A retrospective cohort study from South Africa showed that 73% of the women with prolonged latent phase of labor were nulliparous [6]. Similarly, an active phase disorder of labor is encountered in about 6% of nulliparous and 2% of multiparous women [1, 7]. Arrest in cervical dilatation is the major contributor, and has been shown to raise the risk of cesarean delivery by about four to six fold [6, 9, 10]. Women with active phase disorder are also at increased risk of oxytocin augmentation, operative vaginal delivery, meconium stained amniotic fluid, postpartum hemorrhage and low Apgar score [11]. A retrospective cohort study has also shown that the success of vaginal delivery (including instrumental) in women with active phase disorder was about 33% [12].

It has been said that prolonged second stage of labor occurs in up to 11% of nulliparous women [13] and its management is a challenge for both laboring women and caregivers [14]. This is because some studies reported that there is an increased risk of maternal morbidity such as perineal trauma, chorioamnionitis and operative vaginal delivery [15] and others reported good perinatal and maternal outcome in the majority of the women provided that no evidence of fetal heartbeat derangement [3, 12]. Other reports concluded that prolonged second stage is associated with a high rate of vaginal delivery and maternal morbidity (increased risk of operative vaginal delivery, maternal blood loss and perineal tear) [2, 13, 16]. But, it is said that the length of the second stage has no effect on neonatal outcome as assessed by Apgar score and admission to the neonatal unit [2, 17]. As a result, the management of the prolonged second stage of labor is an unsettled issue, although the common practice is to intervene after a maximum of two hour stagnation.

On the other hand, obstructed labor is reported as an important cause of maternal and perinatal deaths in a community in which operative deliveries are inaccessible [3, 18, 19]. The incidence of obstructed labor in Jimma studies was 7% -12.2% and the commonly attributed cause was cephalopelvic disproportion [20, 21].

In Ethiopia, labor abnormality is expected to be highly prevalent taking into account the high prevalence of obstructed labor and obstetric fistula [20–22]. However, though abnormal labor is known to be one of the most common intrapartum problems encountered in health facilities on a daily basis, the authors could not find a published article from this country that assessed its predictors. Therefore, this case control study could shed some light on potential risk factors associated with labor abnormality. The purpose of this study was to determine predictors of labor abnormality among women who gave birth in the study hospital.

## Methods

### Study setting

An unmatched case control study was conducted by including women who delivered at Hawassa University Hospital between January 2010 and December 2011. The study site serves as a central referral hospital for health facilities in the Southern Region of Ethiopia and two neighboring zones of Oromia region. During the two year study period, a total of 4267 women gave birth in the study hospital.

In the study hospital, each laboring woman was clinically assessed by a midwife, intern physician, different level of postgraduate students, general practitioner and gynecologist. However, the data sources for this study were the clinical findings and decisions made by the most senior person (general practitioner or gynecologist).

### Operational definitions

In this study, labor abnormality or abnormal labor includes prolonged latent phase, active phase disorder, prolonged second stage and obstructed labor. Prolonged latent phase of the first stage of labor was defined when true labor lasting longer than 20 hours for newly paras women and 14 hours for multiparous to enter an active first stage of labor [1]. In the hospital management protocol, active phase disorder includes protracted cervical dilatation (<1 cm/hour cervical dilatation), protracted descent (<1 cm/hour descent of the presenting part), arrest of cervical dilatation (no change in cervical dilatation within 2 hours) and arrest of descent (no change in station within 1 hour). Prolonged second stage of labor was defined when the second stage lasts more than 2 hours for primigravida and more than 1 hour for multiparous women. Administering epidural anesthesia or systemic narcotic was not practiced in any of the laboring mother.

Obstructed labor was diagnosed when a laboring mother presented with > 24 hours of labor, unable to support herself or unable to move her lower extremities, with deranged vital signs, distended bladder, Bandle’s ring formed in the lower uterine segment, fetal distress or death, edematous vulva, big caput, significant molding, foul smelling and thick meconium stained amniotic fluid. Inadequate pelvis was considered when the team leader (general practitioner or gynecologist) assessed the laboring women as a case of cephalopelvic disproportion secondary to contracted pelvis. Inadequate uterine contraction was defined as the frequency was < 3 in 10 min or the duration of contraction lasted < 40 seconds. Since the intensity of uterine contraction had been assessed using fingers, which is very subjective, it was not given credit for the analysis of this study. Gestational ages were proximated to weeks.

### Sample size and sampling

In this analysis, a total of 844 (408 cases and 436 controls) women were included. Cases were laboring mothers who were diagnosed to have one of the types of labor abnormalities (prolonged latent, active phase disorder, prolonged second stage or obstructed labor) at term regardless of mode of delivery and fetal outcome. Controls were mothers who were not diagnosed to have any type of labor abnormality at term regardless of mode of delivery and fetal outcome. For analysis of predictors for the overall labor abnormalities, all cases and controls were included. In the subgroup analysis (specific type of labor abnormalities), the ratio of cases to controls was 1:2.3 in women with active phase disorder, 1:9.3 in women with prolonged latent first stage of labor, 1:5.5 in women with prolonged second stage and 1:6.7 in women with obstructed labor.

The sample size was calculated using Epi info version 2002 by applying the following assumptions: power 80, ratio 1:1, a 1.75 odds ratio of occiputo posterior position as one of the leading causes of labor abnormality with 95% CI. Women who gave birth to singleton at term were included till estimated sample size was fulfilled. Mothers with intrauterine fetal death before the onset of labor, multiple pregnancy, preterm labor, anomalous fetus and those who gave birth by elective cesarean section were not included in this analysis. Since breech presentation at or near term was an absolute indication for caesarean section in the study hospital, all fetuses with breech presentation were excluded.

Using the identification card/chart number of mothers recorded in the delivery log book as a sample frame, systematic sampling method (K^th^ = 5) was used to select the study participants for both cases (any type of labor abnormality) and controls. Since both cases and controls were found recorded in the same log book, equal chance of selection was given. When the randomly selected card number was identified as not eligible for the set inclusion criteria, it was replaced with another one. Charts of selected mothers were retrieved from the hospital record office and were cross checked with the delivery log book.

### Data collection and analysis

Any of the labor abnormality was considered as the dependent variable. Age of the mother, parity, fetal weight, fetal position, inadequate uterine contraction, rupture of fetal membranes, antenatal care, low Apgar score, meconium stained amniotic fluid, and inadequate pelvis were some of the independent variables.

After checking for the completeness, data were entered into SPSS version 16 computer software. Histogram was used for univaribale analysis of the frequency distribution of the different types of labor abnormalities. Bivariable and multivariable logistic regression models were used to test the association of the independent variables with the outcome/dependent variable. All the variables tested in the bivariable analyses were also included in the multivariable analysis to assess the strength of association of those variables which demonstrate statistical significance in the multivariable analysis. Crude and adjusted odds ratios were determined for potential factors associated with one or more types of labor abnormality. Associations were considered statistically significant if the 95% confidence interval of the odds ratio did not include the null value (i.e. 1).

### Ethical approval

Ethical clearance was given by Hawassa University College of Medicine and Health Sciences Institutional Review Board (IRB). Data was collected after a letter of permission was written by the medical director of the hospital’s record office and Obstetrics/Gynaecology department. Additionally, confidentiality and anonymity were assured by analyzing and disseminating the data in aggregate. Since the study was retrospective by design, written informed consent was not obtained from the patient for the publication of this report.

This study has adhered to the international, collaborative initiative of epidemiologists, methodologists, statisticians, researchers and journal editors (STROBE) guidelines.

## Results

**Figure 1:**
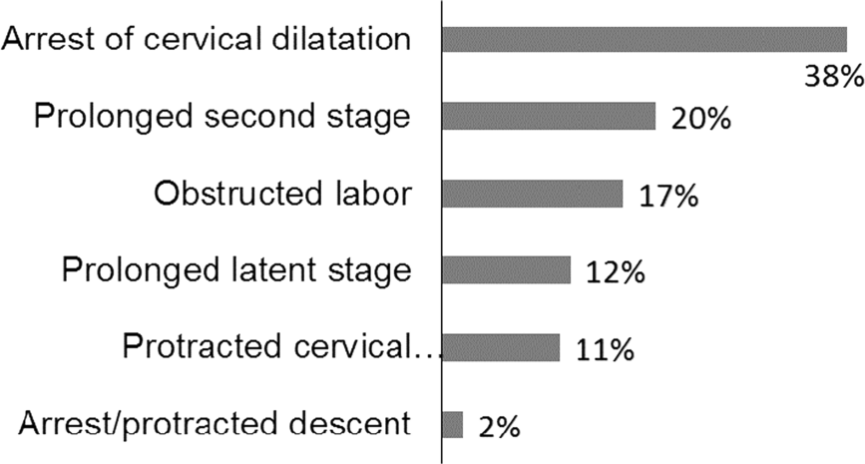
**Types of labor abnormality (cases) at Hawassa university referral hospital/Ethiopia, January 2010-December 2011.** N = 428.

As Figure 1 shows, the majority of labor abnormalities (cases) were arrest of cervical dilatation. Active phase disorder (arrest and protracted cervical dilatation), prolonged second stage and prolonged latent first stage of labor each contributed for 208 (48.6%), 87 (20.3%), and 52 (12.1%) of all types of labor abnormalities. Of the total cases, 72 (16.8%) were obstructed labor.

The mean maternal age and mean gestational age of cases and controls was almost comparable, 24.8 ± 4.8 years vs 24.9 ± 4.9 years, and 38.9 ± 1.9 weeks vs 39 ± 1.9 weeks, respectively. About 80% of both cases and controls had antenatal care. The majority of study participants (59.6% of the cases and 66.1% of controls) were from urban areas. Specific to obstructed labor, 47 (65.3%) came from rural areas. About two-thirds of the cases and controls were primiparous women. More than a quarter of women (both cases and controls) were admitted to the delivery room in the second stage of labor. But it was noted that more controls than cases came to the hospital in a latent first stage of labor. The mean newborns’ birth weight of cases and controls was very comparable (3.3 ± 0.67 kg and 3.3 ± 0.68 kg, respectively). The first and fifth minutes Apgar scores of the majority of cases and controls were also comparable.

Cesarean section and instrumental vaginal delivery were done in 275 (67.4%) and 42 (10.3%) cases, respectively. Out of 275 cesarean section, 81 (29.5%), 111 (40.4%) and 83 (30.2%) were done in latent phase, active phase and second stage of labor, respectively. Neonates delivered after a prolonged latent phase of labor were about six times more likely to have thick meconium stained amniotic fluid than the controls. Intrapartal fetal and early neonatal deaths were 20 (4.2%) among controls and 16 (3.7) among cases. The majority of deaths among cases were attributed to obstructed labor (10/16). However, there was no maternal death among cases or controls.

As presented in Table 1, the logistic regression model did not demonstrate statistically significant association of the overall labor abnormality with maternal age, area of residence, parity, gestational age, uterine contraction, fetal position, fetal membranes and fetal birth weight. Among included, contracted pelvis was the only variable which showed an independent association with the overall labor abnormality (Adjusted OR, 0.5; 95% CI, 0.31-0.79).

**Table 1:** **Logistic regression analysis of the overall labor abnormality at Hawassa referral hospital/Ethiopia, January 2010-December 2011**

Variables	Cases no (%)	Controls no (%)	Crude OR (95%CI)	Adjusted OR (95%CI)*
**Maternal age (yrs):**				
20–34	366(85.5)	399(83.1)	1	1
15–19	34(8.0)	46(9.6)	0.8(0.51–1.28)	0.7(0.46–1.22)
35+	28(6.5)	35(7.3)	0.9(0.52–1.46)	0.9(0.52–1.64)
**Residence:**				
Urban	257(60.0)	305(63.5)	1.2(0.89-1.52)	1.1(0.83–1.50)
Rural	171(40.0)	175(3.5)	1	1
**Parity:**				
Multiparous	115(26.9)	140(29.2)	1	1
Grandmultiparous	30(7.0)	33(6.9)	0.9(0.66-1.20)	0.9(0.65–1.22)
Primiparous	283(66.1)	307(63.9)	1.0(0.59-1.66)	0.9(0.52–1.69)
**Gestational age (wks):**				
37–41	350(81.8)	403(84.0)	1	1
28–36	11(2.6)	13(2.7)	0.9(0.43–2.20)	1.1(0.45–2.61)
42+	67(15.6)	64(13.3)	1.2(0.83–1.75)	1.1(0.75–1.61)
**Uterine contraction:**				
Adequate	204(47.7)	243(50.6)	1	1
Inadequate	224(52.3)	237(49.4)	1.1(0.87–1.46)	1.1(0.86–1.49)
**Pelvic status:**				
Adequate	352(82.3)	365(76.0)	1	1
Contracted	45(10.5)	46(9.6)	1.0(0.66–1.57)	1.0(0.66–1.63)
Unknown	31(7.2)	69(14.4)	0.5(0.30–1.73)	0.5(0.31–0.79)**
**Fetal membranes:**				
Intact	95(22.2)	112(23.3)	0.9(0.68–1.28)	1.0(0.72–1.43)
PROM	28(6.5)	37(7.7)	0.9(0.51–1.46)	0.9(0.54–1.54)
Ruptured during labor	302(70.6)	331(69.0)	1	1
**Fetal Position:**				
Occipitoanterior	88(20.6)	93(19.4)	1	1
Occipito posterior	60(14.0)	62(12.9)	1.0(0.65–1.62)	1.0(0.62–1.62)
Occipito transverse	100(23.4)	98(20.4)	1.1(0.72–1.61)	1.0(0.69–1.58)
Other	180(42.0)	227(47.3)	0.8(0.59–1.19)	0.9(0.60–1.26)
**Birth weight (gram):**				
2500–3999	352(82.2)	406(84.6)	1	1
<2500	12(2.8)	10(2.1)	1.4(0.59–3.24)	1.4(0.58–3.38)
4000+	55(12.9)	55(11.4)	1.2(0.77–1.72)	1.1(0.72–1.65)
Unknown	9(2.1)	9(1.9)	1.2(0.45–2.94)	1.3(0.48–3.31)

*Adjusted for all the variables in this Table. **P < 0.01.

As shown in Tables 2, 3, 4, 5, however, the subgroup analyses by specific type of labor abnormality have shown statistically significant associations with some more variables. In Table 2 (adjusted logistic regression analysis), it was found that grand multiparous women were about 60% less likely to have a prolonged latent first stage of labor. Women with postterm pregnancy (AOR, 2.3; 95% CI, 01.07-4.96), inadequate uterine contraction (AOR, 2.3; 95% CI, 1.15-4.68) and intact fetal membranes (AOR, 6.9; 95% CI, 3.39-14.17) or premature rupture of fetal membranes (AOR, 4.5; 95% CI, 1.42-14.19) were independent predictors of prolonged latent first stage of labor.

**Table 2:** **Logistic regression analysis of prolonged latent phase of labor at Hawassa referral hospital/Ethiopia, January 2010-December 2011**

Variables	Cases no (%)	Controls no (%)	Crude OR (95%CI)	Adjusted OR (95%CI) ^‡^
**Maternal age (years):**				
20–34	47(90.4)	399(83.1)	1	1
15–19	3(5.8)	46(9.6)	0.4(0.08–1.54)	0.3(0.06–1.38)
35+	2(3.8)	35(7.3)	0.5(0.11–2.01)	0.9(0.17–5.23)
**Residence:**				
Urban	43(82.7)	305(63.5)	2.7 (1.31–3.76)**	2.0(0.88–4.73)
Rural	9(17.3)	175(3.5)	1	1
**Parity:**				
Multiparous	9(17.3)	140(29.2)	1	1
Grandmultiparous	2(3.8)	33(6.9)	0.5(0.23–1.02)	0.4(0.17–0.89)*
Primiparous	41(78.9)	307(63.9)	0.5(0.11–1.96)	0.7(0.13–4.27)
**Gestational age(weeks):**				
37–41	36(69.2)	403(84.0)	1	1
28–36	1(1.9)	13(2.7)	0.9(0.13–6.77)	0.8(0.09–8.01)
42+	15(28.9)	64(13.3)	2.6(1.36–5.06)**	2.3(1.07–4.96)**
**Uterine contraction:**				
Adequate	15(28.9)	243(50.6)	1	1
Inadequate	37(71.1)	237(49.4)	2.5(1.35–4.73)**	2.3(1.15–4.68)*
**Pelvic status:**				
Adequate	44(84.6)	365(76.0)	1	1
Contracted	3(5.8)	46(9.6)	0.5(0.16–1.81)	0.8(0.20–3.12)
Unknown	5(9.6)	69(14.4)	0.6(0.23–1.65)	0.5(0.19–1.54)
**Fetal membranes:**				
Intact	34(65.4)	112(23.3)	7.8(3.96–5.27)^†^	6.9(3.39–14.17)†
PROM	5(9.6)	37(7.7)	5.6(1.21–10.67)*	4.5(1.42–14.19)*
Ruptured during labor	13(25.0)	331(69.0)	1	1
**Birth weight (gram):**				
2500–3999	43(82.7)	406(84.6)	1	1
<2500	2(3.8)	10(2.1)	1.9(0.40–8.90)	2.0(0.37–11.64)
4000+	6(11.6)	55(11.4)	1.0(0.42–2.53)	1.0(0.39–3.02)
Unknown	1(1.9)	9(1.9)	1.0(0.13–8.48)	4.9(0.51–46.72)

^‡^Adjusted for all the variables in this Table. *P < 0.05; **P < 0.01, ^†^P < 0.0001.

**Table 3:** **Logistic regression analysis of active phase disorder at Hawassa referral hospital/Ethiopia, January 2010-December 2011**

Variables	Cases no (%)	Controls no (%)	Crude OR (95%CI)	Adjusted OR (95%CI) ^‡^
**Maternal age (yrs):**				
20–34	175(84.1)	399(83.1)	1	1
15–19	21(10.1)	46(9.6)	1.0 (0.60–1.79)	1.0(0.54–1.77)
35+	12(5.8)	35(7.3)	0.8 (0.39–1.54)	0.9(0.43–1.98)
**Residence:**				
Urban	130(62.5)	305(63.5)	1.0 (0.68–1.34)	0.9(0.63–1.33)
Rural	78(37.5)	175(3.5)	1	1
**Parity:**				
Multiparous	53(25.4)	140(29.2)	1	1
Grandmultiparous	13(6.3)	33(6.9)	0.8(0.56–1.19)	0.8(0.57–1.29)
Primiparous	142(68.3)	307(63.9)	0.9(0.44–1.67)	0.9(1.41–1.94)
**Gestational age(wks):**				
37–41	172(82.7)	403(84.0)	1	1
28–36	4(1.9)	13(2.7)	0.7(0.23–2.24)	0.7(0.20–2.26)
42+	32(15.4)	64(13.3)	1.2(0.74–1.86)	1.0(0.65–1.70)
**Uterine contraction:**				
Adequate	81(38.9)	243(50.6)	1	1
Inadequate	127(61.1)	237(49.4)	1.6(1.15–2.24)**	1.5(1.06–2.13)*
**Pelvic status:**				
Adequate	188(90.4)	365(76.0)	1	1
Contracted	9(4.3)	46(9.6)	0.4(0.18–0.79)**	0.5(0.21–0.95)*
Unknown	11(5.3)	69(14.4)	0.3(0.16–0.60)^†^	0.4(0.19–0.74)**
**Fetal membranes:**				
Intact	55(26.4)	112(23.3)	1.2(0.82–1.75)	1.3(0.84-1.97)
PROM	15(7.2)	37(7.7)	1.0(0.54–1.93)	1.1(0.54–2.05)
Ruptured during labor	138(66.4)	331(69.0)	1	1
**Fetal Position:**				
Occipitoanterior	63(30.3)	93(19.4)	1	1
Occipito posterior	21(10.1)	62(12.9)	0.5(0.28–0.90)*	0.6(0.32–1.07)
Occipito transverse	50(24.0)	98(20.4)	0.8(0.47–1.20)	0.8(0.51–1.34)
Other	74(35.6)	227(47.3)	0.5(0.32–0.73)**	0.5(0.34–0.81)**
**Birth weight (gram):**				
2500–3999	177(85.1)	406(84.6)	1	1
<2500	7(3.4)	10(2.1)	1.6(0.60–4.28)	1.7(0.59-4.63)
4000+	23(11.0)	55(11.4)	0.9(0.57–1.61)	1.0(0.55–1.66)
Unknown	1(0.5)	9(1.9)	0.3(0.03–2.03)	0.4(0.05–3.08)

^‡^Adjusted for all the variables in this Table. *P < 0.05; **P < 0.01, ^†^P < 0.0001.

**Table 4:** **Logistic regression analysis of prolonged second stage of labor at Hawassa referral hospital/Ethiopia, January 2010-December 2011**

Variables	Cases no (%)	Controls no (%)	Crude OR (95% CI)	Adjusted OR (95% CI) ^‡^
**Maternal age (yrs):**				
20–34	76(87.4)	399(83.1)	1	1
15–19	5(5.7)	46(9.6)	0.6 (0.22–1.48)	0.6(0.31–1.78)
35+	6(6.9)	35(7.3)	0.9 (0.37–2.21)	1.1(0.39–2.99)
**Residence:**				
Urban	54(62.1)	305(63.5)	0.9 (0.59–1.50)	1.1(0.64–1.82)
Rural	33(37.9)	175(3.5)	1	1
**Parity:**				
Multiparous	28(32.2)	140(29.2)	1	1
Grandmultiparous	5(5.7)	33(6.9)	1.1(0.69–1.87)	1.1(0.61–1.84)
Primiparous	54(62.1)	307(63.9)	0.9(0.32–2.30)	0.8(0.25–2.42)
**Gestational age(wks):**				
37–41	71(81.6)	403(84.0)	1	1
28-36	3(3.5)	13(2.7)	1.3(0.36–4.71)	1.8(0.41–7.56)
42+	13(14.9)	64(13.3)	1.2(0.60–2.20)	1.2(0.61–2.42)
**Uterine contraction:**				
Adequate	57(65.5)	243(50.6)	1	1
Inadequate	30(34.5)	237(49.4)	0.5(0.34–0.87)*	0.6(0.35–0.97)*
**Pelvic status:**				
Adequate	72(82.8)	365(76.0)	1	1
Contracted	4(4.6)	46(9.6)	0.4(0.15–1.26)	0.4(0.14–1.20)
Unknown	11(12.6)	69(14.4)	0.8(0.41–1.60)	1.0(0.47–2.20)
**Fetal membranes:**				
Intact	4(4.6)	112(23.3)	0.2(0.06–0.43)^†^	0.2(0.07–0.54)**
PROM	4(4.6)	37(7.7)	0.5(0.17–1.39)	0.5(0.17–1.53)
Ruptured during labor	79(90.8)	331(69.0)	1	1
**Fetal Position:**				
Occipitoanterior	18(20.7)	93(19.4)	1	1
Occipito posterior	17(19.5)	62(12.9)	1.4(0.68–2.96)	1.1(0.51–2.48)
Occipito transverse	26(29.9)	98(20.4)	1.4(0.71–2.66)	1.3(0.63–2.57)
Other	26(29.9)	227(47.3)	0.6(0.31–1.13)	0.7(0.34–1.34)
**Birth weight (gram):**				
2500–3999	69(79.3)	406(84.6)	1	1
<2500	2(2.3)	10(2.1)	1.2(0.25–5.49)	1.1(0.19–6.08)
4000+	15(17.3)	55(11.4)	1.6(0.85–2.99)	1.6(0.80–3.12)
Unknown	1(1.1)	9(1.9)	0.7(0.08–5.24)	0.6(0.07–5.27)

^‡^Adjusted for all the variables in this Table. * P < 0.05; ** P < 0.01, ^†^P < 0.0001.

**Table 5:** **Logistic regression analysis of obstructed labor at Hawassa referral hospital/Ethiopia, January 2010-December 2011**

Variables	Cases no (%)	Controls no (%)	Crude OR (95%CI)	Adjusted OR 95%CI) ^‡^
**Maternal age (yrs):**				
20–34	59(82.0)	399(83.1)	1	1
15–19	6(8.3)	46(9.6)	0.9 (0.36–2.16)	0.8(0.29–2.31)
35+	7(9.7)	35(7.3)	1.4 (0.57–3.18)	0.7(0.24–2.13)
**Residence:**				
Urban	25(34.7)	305(63.5)	1	1
Rural	47(65.3)	175(3.5)	3.3 (1.95–5.51) †	2.0(1.10–3.76)*
**Parity:**				
Multiparous	23(31.9)	140(29.2)	1	1
Grandmultiparous	9(12.5)	33(6.9)	1.3(0.73–2.19)	1.4(0.69–2.63)
Primiparous	40(55.6)	307(63.9)	2.1(0.93–4.69)	1.8(0.65–4.98)
**Gestational age(wks):**				
37–41	65(90.3)	403(84.0)	1	1
28–36	2(2.8)	13(2.7)	0.9(0.21–4.33)	1.1(0.14–8.66)
42+	5(6.9)	64(13.3)	0.5(0.19–1.25)	0.6(0.21–1.61)
**Antenatal are**				
Yes	47(65.3)	378(78.7)	1	1
No	20(27.8)	58(12.1)	2.8(1.54–5.01)**	2.1(1.04–4.42)*
Unknown	5(6.9)	44(9.2)	0.5(0.35–2.42)	1.1(0.37–3.25)
**Uterine contraction:**				
Adequate	46(63.9)	243(50.6)	1	1
Inadequate	26(36.1)	237(49.4)	0.6(0.35–0.97)*	0.7(0.39–1.28)
**Pelvic status:**				
Adequate	44(61.1)	365(76.0)	1	1
Contracted	25(34.7)	46(9.6)	4.5(2.53-8.04) †	2.8(1.47–5.41)**
Unknown	3(4.2)	69(14.4)	0.4(0.11-1.19)	0.3(0.07–1.51)
**Fetal membranes:**				
Intact	2(2.8)	112(23.3)	0.1(0.02–0.36)**	0.1(0.03–0.59)**
PROM	2(2.8)	37(7.7)	0.1(0.02–1.02)	0.2(0.03–1.64)
Ruptured during labor	68(94.4)	331(69.0)	1	1
**Fetal Position:**				
Occipitoanterior	2(2.8)	93(19.4)	1	1
Occipito posterior	19(26.4)	62(12.9)	14.3(3.21–63.36)^†^	9.7(2.03–45.98)**
Occipito transverse	16(22.2)	98(20.4)	7.6(1.70–33.92)**	5.0(1.04–23.55)*
Other	35(48.6)	227(47.3)	7.2(1.69–30.42)**	7.6(1.70–34.27)**
**Birth weight (gram):**				
2500–3999	56(77.8)	406(84.6)	1	1
<2500	1(1.4)	10(2.1)	0.7(0.09–5.77)	1.1(0.11–10.72)
4000+	9(12.5)	55(11.4)	1.2(0.56–2.53)	0.8(0.32–1.83)
Unknown	6(8.3)	9(1.9)	4.8(1.66–14.09)**	2.8(0.76–10.37)

^‡^Adjusted for all the variables in this Table. * P < 0.05; ** P < 0.01, ^†^P < 0.0001.

As presented in Table 3, in adjusted analysis, it was found that women with inadequate uterine contraction were about 1.5 times more likely to have the active phase disorder than those with adequate uterine contraction. Laboring women with contracted pelvis were about 50% more likely to have the active phase disorder. In unadjusted odds ratio, fetuses with occipito posterior position were about 50% more likely to have the active phase disorder than fetuses with occipito anterior position.

Table 4 shows that in the adjusted analysis, the two independent predictors prolonged second stage of labor were inadequate uterine contraction and intact fetal membranes. Both crude and adjusted logistic regression model did not demonstrate a statistically significant association of prolonged second stage with maternal age, parity, pelvic status, fetal position and fetal birth weight.

As shown in Table 5, the adjusted odds ratio revealed that women from rural areas were about 2-fold more likely to have obstructed labor than those who came from urban areas. Lack of antenatal care was also strongly associated with obstructed labor (AOR, 2.1; 95% CI, 1.04-4.42). Women with contracted pelvis were 2.8 times more likely to have obstructed labor. Both occipito posterior (AOR 9.7; 95% CI, 2.03-45.98) and occipito transverse (AOR, 5.0; 95% CI, 1.04-23.55) fetal positions were independent predictors of obstructed labor.

## Discussion

This study has demonstrated that the active phase disorder accounted for nearly half of the total cases included in this analysis. The fact that most cases had antenatal care and came from urban areas (with the exception of obstructed labor cases) has probably facilitated early diagnosis and management in many mothers, before they developed obstructed labor. In other words, the majority of obstructed labor cases came from rural areas and had no antenatal care follow up, which indicate the significant delay in either health care seeking behavior or getting access to a health facility starting from pregnancy. The high neonatal death among obstructed labor cases is also another evidence for the long delay in terms of providing appropriate intervention during the early stages of labor abnormality. It is known that the high perinatal mortality due to obstructed labor are usually attributed to intrapartum asphyxia, which can be averted by timely performing operative deliveries [13, 20, 21].

The finding of more than one-fourth of cases and nearly one third of controls being in the second stage of labor at the time of admission also signifies the big delay to come to the hospital. What matters for the diagnosis of early stage labor abnormalities or to the extreme obstructed labor at the time of admission was probably the total duration of stay either at home or somewhere in other health facility. It is a known fact that the longer the delay in the second stage of labor, the more likely to develop obstructed labor and its complications [13].

Unlike previous reports [1, 9], however, the commonly attributed risk factors for poor progress of labor (maternal age < 20 years or above 34 years and macrosomia) were not found to have a statistically significant association with any of the labor abnormalities including obstructed labor. Furthermore, nulliparity and post term pregnancy were not found to have a statistically significant association with the active phase disorder, prolonged second stage and obstructed labor. This might be due to the big delay observed in both cases and controls.

In other words, this study compared cases (labor abnormalities) and controls (normally progressing labor) which somehow all came to the study hospital from the same community and likely to have comparable health seeking behavior. As a result, some normal deliveries which were categorized as controls might already have one type of labor abnormality, which were probably overlooked because of multiple reasons. To begin with, those who came in the second stage and had a normal delivery might already have a latent or active phase disorder while at home or somewhere on the way. Secondly, since the diagnosis of labor abnormality was based on the change in cervical dilatation and descent (as assessed by digital pelvic examination) in a specific period of time, which is very subjective and liable to inter- and intra-observer variation, there was a chance to diagnose a normally progressing labor as labor abnormality. The other side of the coin is that since some women with labor abnormality were probably given additional time and delivered normally, they were grouped as controls.

Another evidence to doubt the precision of digital pelvic assessment during the study period is the finding of about 82% of the pelvic status of women with labor abnormalities was reported as adequate. This finding probably implies that there is likely to have a significant gap among managing health professionals in terms of assessing the pelvic architecture. This is likely to happen since there were several general practitioners and gynecologists as a final decision maker. Equally important is the high intrapartum and early neonatal deaths in the control group (4.2% vs 3.7%), in which some form of labor abnormalities might have contributed to increased fetal losses in the control group.

To be more specific, prolonged latent first stage of labor, active phase disorder, prolonged second stage of labor and obstructed labor were the common types of labor abnormalities reported. Many studies showed that prolonged latent first stage of labor was associated with maternal age >35 years, nulliparity, occipito posterior fetal position, fetal birth weight > 4 kg, and meconium stained amniotic fluid [1, 2, 8, 9]. In this study and some other studies [8, 6, 23], however, prolonged latent phase of labor showed no association with maternal age, birth weight, and pelvic status. This is probably because the latent phase disorder is a phenomenon encountered before the cervix is fully effaced, in which the size of the fetus and the adequacy of the pelvis are likely to have little effect on latent phase labor progress. It should also be noted that a highly significant association of prolonged latent phase of labor with inadequate uterine contraction was similar to other studies [8, 10]. There was no association between prolonged latent phase and parity, which was consistent with another report [24].

In this study, fetal malpositions had no association with the active phase disorder, which is inconsistent with other studies [2, 10] but consistent with Myles et al. study [15]. Furthermore, partly consistent with Myles et al. but contradictory to Janni et al. [16] study findings, this study did not demonstrate an association of active phase disorder with maternal age, parity, gestational age and birth weight. Since all study participants included in this study were at term (37+ weeks), a difference of few weeks may have little effect on the progress of active first stage of labor. Furthermore, since the fetal head size and position are known to determine the descent, which is usually expected to occur more in the second stage of labor [1, 5], the lack of association of birth weight and position with the active phase disorder may be realistic. However, multiparous women are known to have a good progress of labor in all stages of labor unless there is a new development in the current pregnancy like persistent malpresentation, malposition or this time big baby. Therefore, the absence of association of active phase disorder with primigravidity is an area of investigation.

On the other hand, previous reports have shown that prolonged second stage of labor has an association with big babies, nulliparity, and occiput posterior position [14, 15, 23], which were not demonstrated in this study and three other studies [15, 20, 25]. The finding of statistically significant association of prolonged second stage of labor with inadequate uterine contraction and intact fetal membranes was not observed in another report [2]. In general, the inconsistent findings on prolonged second stage association with parity, fetal weight and position need further investigation, preferably using prospective cohort design.

This study has shown an independent association of obstructed labor with rural residence, lack of antenatal care, contracted pelvis and fetal head malpositions, which is similar to other reports [4, 13, 21]. The lack of antenatal care and the development of obstructed labor by itself (which usually occurs among skilled personnel unattended home deliveries) may show the big delay in deciding to seek care. Furthermore, being the majority of the women with obstructed labor from the rural area is another evidence for the delay in arriving at a health facility that provides a comprehensive obstetric service.

In general, although there were several laboring women who came in the second stage from urban areas, a 2-fold increased risk of obstructed labor among women from rural area may be taken as a strong predictor for obstructed labor, which was also reported by other investigators [4, 20, 21]. Therefore, apart from creating awareness on the whole package of medical service to pregnant women, regardless of area of residence, availing the comprehensive obstetric service for the rural women in the study area may reduce or avert the occurrence of obstructed labor and its sequel.

As partly discussed above, this study has several limitations. Since the majority of the rural women have a habit of laboring and delivering at home, the sampled study participants were mainly from urban areas that could not be representative of the general population in the study area. Secondly, there is a possibility of low precision in making diagnosis of labor abnormality because of the nature of subjective assessment of labor progress (inter- and intra-observer variations). Thirdly, because of the little but progressive change as the duration of labor advances and because of the significant delay observed in both cases and controls, some of the cases could have been diagnosed at an early stage of labor and some of the controls could have been diagnosed as cases if they had reported to the hospital before the labor advanced to a late stage. Fourthly, the retrospective nature of the study was also a limitation to perform further analysis by including other variables.

## Conclusion

More than a quarter of cases and controls came to the hospital in the second stage of labor. Cesarean delivery was the most common type of intervention undertaken in cases. The active phase disorder was the commonest type of labor abnormality. The logistic regression analysis demonstrated an independent association of overall labor abnormality with pelvic inadequacy. Malposition, inadequate pelvis and inadequate uterine contraction were some of the independent predictors of specific types of labor abnormalities. The barriers for laboring women not to come to a health facility in time is an area of investigation. Specifically, since the majority of women with obstructed labor came from a rural area and had no antenatal care follow up, health promotion to the rural community in terms of awareness creation on potential complications during labor and delivery is at the forefront.
